# Supplementary material for: Experimental violation of n-locality in a star quantum network
Source: Nat Commun. 2020 May 18;11:2467. doi: 10.1038/s41467-020-16189-6 (PMC7235259; doi:10.1038/s41467-020-16189-6)
Supplement: Supplementary file 1 — Supplementary Information [file 41467_2020_16189_MOESM1_ESM.pdf]

Supplementary Information for
Experimental violation of n-locality in a star quantum network

Poderini et al.

Supplementary Note 1. PHOTON SOURCES

In our experiment, we exploited four different photon sources generating polarization entangled photon pairs in the singlet state[1]: $|\psi^-\rangle = (|01\rangle - |10\rangle)/\sqrt{2}$ through a type-II Spontaneous Parametric Down-Conversion (SPDC) process (see Supplementary Figure 5). Within three laboratories (Laboratory 1, 3 and 4), the generation was carried out by pumping 2 mm-thick Beta-Barium Borate (BBO) crystals with a Ti:Sapphire mode locked laser, with repetition rate of 76 MHz. The generation in Lab 2, instead, was accomplished with a continuous wave diode laser which pumped a periodically-poled KTP crystal inside a Sagnac interferometer[2, 3]. One photon of each laboratory A_i was sent via optical single-mode fibers to the central measurement station B while the other was locally measured within the original laboratory. The quality of the generated singlet states was verified by performing a CHSH test for each pair, whose results are reported in Supplementary Table 1. Let us note that each Laboratory employs a different and independent power supply system to generate the laser pump for their source.

A. Laboratory 1

Photon pairs were generated by a 2 mm-thick BBO crystal pumped by a pulsed field with $\lambda = 392.5$ nm. The detected coincidence rate was 25 000 Hz, while a CHSH inequality violation extent of $S^{(\text{raw})} = 2.408 \pm 0.018$ was observed.

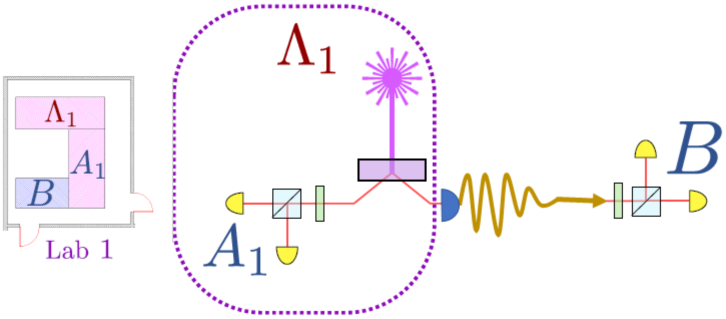

Supplementary Figure 1: **Schematic representation of Laboratory 1.** Polarization-entangled photon pairs are generated via type-II Spontaneous Parametric Down-Conversion (SPDC) in a BBO crystal (Λ_1). The generated photons are spectrally filtered, via a narrow band interference filter ($\Delta\lambda = 3$ nm), and in the spatial mode, via a single-mode fiber. One of the photons is directly measured in the laboratory where it was generated, within a local measurement station A_1, while the other is sent through an optical fiber to the central measurement station B. Both photons are locally measured in the corresponding station by exploiting a half-wave plate, rotated by an angle θ, and a polarizing beam splitter, which allows to perform polarization projective measurements of the following form: $\cos(4\theta)\sigma_z + \sin(4\theta)\sigma_x$.

B. Laboratory 2

Photon pairs were generated by a 20mm-long ppKTP crystal pumped by a single longitudinal mode continuous wave $\lambda = 404$ nm laser. The detected coincidence rate was 45 000 Hz, while a CHSH inequality violation extent of $S^{(\text{raw})} = 2.395 \pm 0.021$. The experimental setup corresponding to this laboratory is depicted in Supplementary Figure 2.

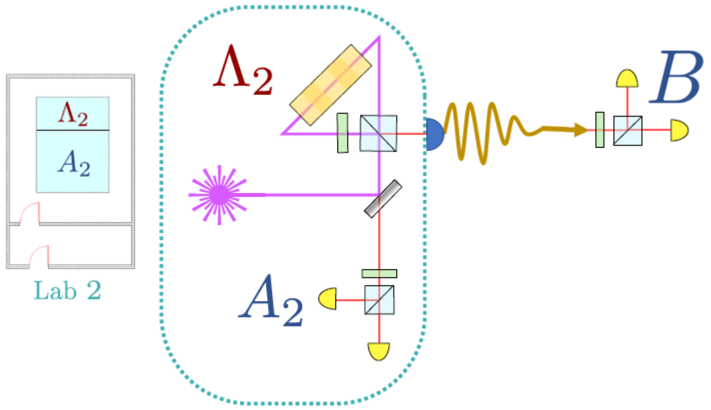

Supplementary Figure 2: **Schematic representation of Laboratory 2.** Polarization-entangled photon pairs are generated via type-II Spontaneous Parametric Down-Conversion (SPDC) in a ppKTP crystal (Λ_2). The generated photons are spectrally filtered, via a narrow band interference filter ($\Delta\lambda = 3$ nm), and in the spatial mode, via a single-mode fiber. One of the photons is directly measured in the laboratory where it was generated, within a local measurement station A_2, while the other is sent through an optical fiber to the central measurement station B. Both photons are locally measured by exploiting a half-wave plate, rotated by an angle θ, and a polarizing beam splitter, which allows to perform polarization projective measurements of the following form: $\cos(4\theta)\sigma_z + \sin(4\theta)\sigma_x$.

C. Laboratory 3

Photon pairs were generated by a 2 mm-thick BBO crystal pumped by a pulsed field with $\lambda = 392.5$ nm. The detected coincidence rate was 1 000 Hz, while a CHSH inequality (see Eq. (5) in the main text) violation extent of $S^{(\text{raw})} = 2.388 \pm 0.020$ was observed. The experimental setup corresponding to this laboratory is depicted in Supplementary Figure 3.

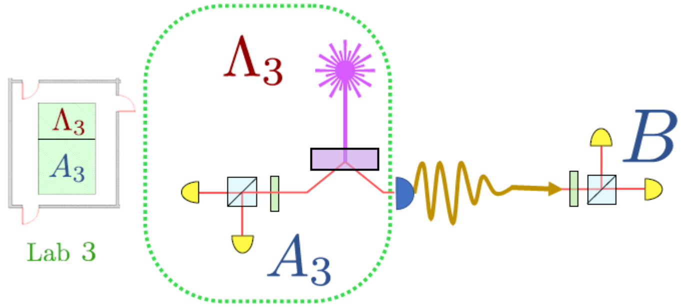

Supplementary Figure 3: **Schematic representation of Laboratory 3.** Polarization-entangled photon pairs are generated via type-II Spontaneous Parametric Down-Conversion (SPDC) in a BBO crystal (Λ_3). The generated photons are spectrally filtered, via a narrow band interference filter ($\Delta\lambda = 3$ nm), and in the spatial mode, via a single-mode fiber. One of the photons is directly measured in the laboratory where it was generated, within a local measurement station A_3, while the other is sent through an optical fiber to the central measurement station B. Both photons are locally measured in the corresponding station by exploiting a half-wave plate, rotated by an angle θ, and a polarizing beam splitter, which allows to perform polarization projective measurements of the following form: $\cos(4\theta)\sigma_z + \sin(4\theta)\sigma_x$.

D. Laboratory 4

Photon pairs were generated by a 2 mm-thick BBO crystal pumped by a pulsed field with $\lambda = 392.5$ nm. The detected coincidence rate was 5 000 Hz, while a CHSH inequality violation extent of $S^{(\text{raw})} = 2.463 \pm 0.013$ was observed. The experimental setup corresponding to this laboratory is depicted in Supplementary Figure 4.

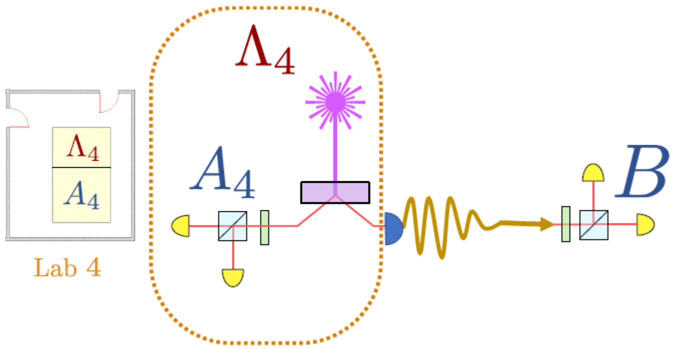

Supplementary Figure 4: **Schematic representation of Laboratory 4.** Polarization-entangled photon pairs are generated via type-II Spontaneous Parametric Down-Conversion (SPDC) in a BBO crystal (Λ_4). The generated photons are spectrally filtered, via a narrow band interference filter ($\Delta\lambda = 3$ nm), and in the spatial mode, via a single-mode fiber. One of the photons is directly measured in the laboratory where it was generated, within a local measurement station A_4, while the other is sent through an optical fiber to the central measurement station B. Both photons are locally measured in the corresponding station by exploiting a half-wave plate, rotated by an angle θ, and a polarizing beam splitter, which allows to perform polarization projective measurements of the following form: $\cos(4\theta)\sigma_z + \sin(4\theta)\sigma_x$.

Source	Crystal	Lab.	λ(nm)	Pump laser	Two-fold CC^1 (Hz)	Experimental $CHSH^1$	Estimated Visibility
Λ_1	BBO	1	785	Pulsed (76 MHz)	25 000	2.408 ± 0.018	0.763 ± 0.010
Λ_2	ppKTP	2	808	Continuous	45 000	2.395 ± 0.021	0.755 ± 0.012
Λ_3	BBO	3	785	Pulsed (76 MHz)	1 000	2.388 ± 0.020	0.751 ± 0.011
Λ_4	BBO	4	785	Pulsed (76 MHz)	5 000	2.463 ± 0.013	0.793 ± 0.007

Supplementary Table 1: **Experimental details for the different laboratories:** In the table are listed the crystals, wavelength, type of pump laser, coincidence rate and experimental violation of the CHSH inequality within each laboratory. The visibilities were obtained by exploiting the noise model and the experimental violation of the CHSH inequality. The superscript ^{1} refers to the average values upon all the collected data.

E. Star-shaped network configuration

By exploiting the four previously described laboratories and the optical fibers connecting them, we are able to implement a star-shaped quantum network consisting of up to five nodes (A_1, A_2, A_3, A_4, B). Indeed, as already mentioned, each laboratory generates a photon pair and locally measures one of the two photons, while the other one is coupled into single mode fibers and is sent to a central node (B). In our case, the central node is located in Lab. 1, see Supplementary Figure 5.

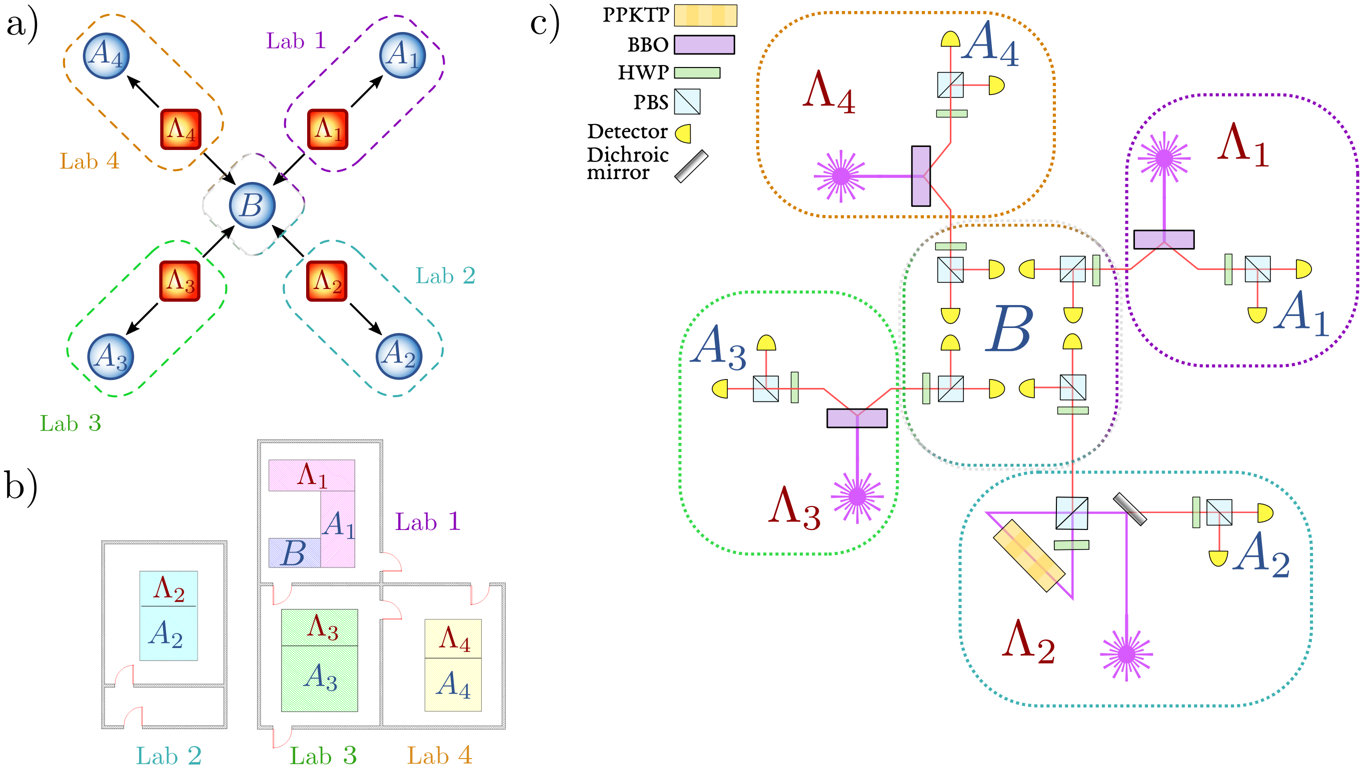

Supplementary Figure 5: **Implementation of a star-shaped network. a)** Spatial configuration of the four different laboratories. Each laboratory has its own single-photon source and one measurement station, composed of a rotated half wave plate and a polarizing beam splitter, in order to perform local polarization projective measurements. The only exception is the central laboratory (Lab 1), which has four separate measurement stations (B), to perform local measurements on all the incoming photons. **b)** Physical location of the laboratories and scheme of the experimental apparatus. Distinct laboratories contain one source and one measurement station each, with the exception of Lab. 1, which also contains the central node B of the star-network on a separate optical table. **c)** Polarization-entangled photon pairs are generated via Spontaneous Parametric Down-Conversion (SPDC) by exploiting four separated non-linear crystals Λ_1, Λ_2, Λ_3 and Λ_4. The peripheral measurement stations A_1, A_2, A_3 and A_4 are located in each laboratory. One photon from each source is sent to the central measurement station B through single-mode optical fibers while the other photon remains within the laboratory where it was generated.

Supplementary Note 2. NOISE MODEL

Different sources of noise must be considered within the experimental implementation. The noise can be taken into account adding decoherence terms when modelling the quantum state shared by the parties.[5] The photon pairs in our experiment are generated in the singlet state ρ_{sing}, that in the base $\{|HH\rangle, |HV\rangle, |VH\rangle, |VV\rangle\}$ can be expressed by the following matrix form:

$$\rho_{\text{sing}} = \frac{1}{2}\begin{bmatrix} 0 & 0 & 0 & 0 \\ 0 & 1 & -1 & 0 \\ 0 & -1 & 1 & 0 \\ 0 & 0 & 0 & 0 \end{bmatrix}. \tag{1}$$

In particular, two different classes of noise affect the SPDC sources [4]:

- White noise, which consists in an isotropic depolarization of the states:

$$\rho_{\text{w}} = v|\psi^-\rangle\langle\psi^-| + (1-v)\frac{\mathbb{I}}{4}, \tag{2}$$

where $\frac{\mathbb{I}}{4}$ is the completely mixed state.

- Colored noise, which is depolarization along a preferred direction, intrinsic of the SPDC generation process, described by the following matrix:

$$M_{\text{colored}} = \frac{1}{2}\begin{bmatrix} 0 & 0 & 0 & 0 \\ 0 & 1 & 0 & 0 \\ 0 & 0 & 1 & 0 \\ 0 & 0 & 0 & 0 \end{bmatrix}. \tag{3}$$

The resulting noisy state, then, is the following:

$$\rho_{\text{c}} = v|\psi^-\rangle\langle\psi^-| + (1-v)M_{\text{colored}} = v|\psi^-\rangle\langle\psi^-| + \frac{(1-v)}{2}(|\psi^-\rangle\langle\psi^-| + |\psi^+\rangle\langle\psi^+|). \tag{4}$$

The final state can be modelled combining these different contributions in a normalized form:

$$\rho = v|\psi^-\rangle\langle\psi^-| + (1-v)[\lambda\frac{|\psi^-\rangle\langle\psi^-| + |\psi^+\rangle\langle\psi^+|}{2} + (1-\lambda)\frac{\mathbb{I}}{4}], \tag{5}$$

where v and λ represent the total noise and the fraction of colored noise, respectively.

A. Noise in the star-network

The effects generated by white and colored noise can be extended to the more complex case of the network, where all of the nodes contain separate noise contributions. In this preliminary analysis, however, we consider that the noise parameters coincide for all of the sources. The more general model, where every source has individual noise parameters, is used in the laboratory-efficiency estimation described in the next section.

The action of both white and colored noises is observed in the reduction of the amount of violation of the chained n-locality inequality (see Eq.(5) in the main text). By comparing quantitatively the two effects for the same noise strength, we observe that white noise provides a larger reduction of S. An example of this behaviour is depicted in Supplementary Figure 6 that shows how white and colored noise affects the experimental violation for the particular case of 4-nodes star-shaped network, where each agent performs $k = 3$ measurements.

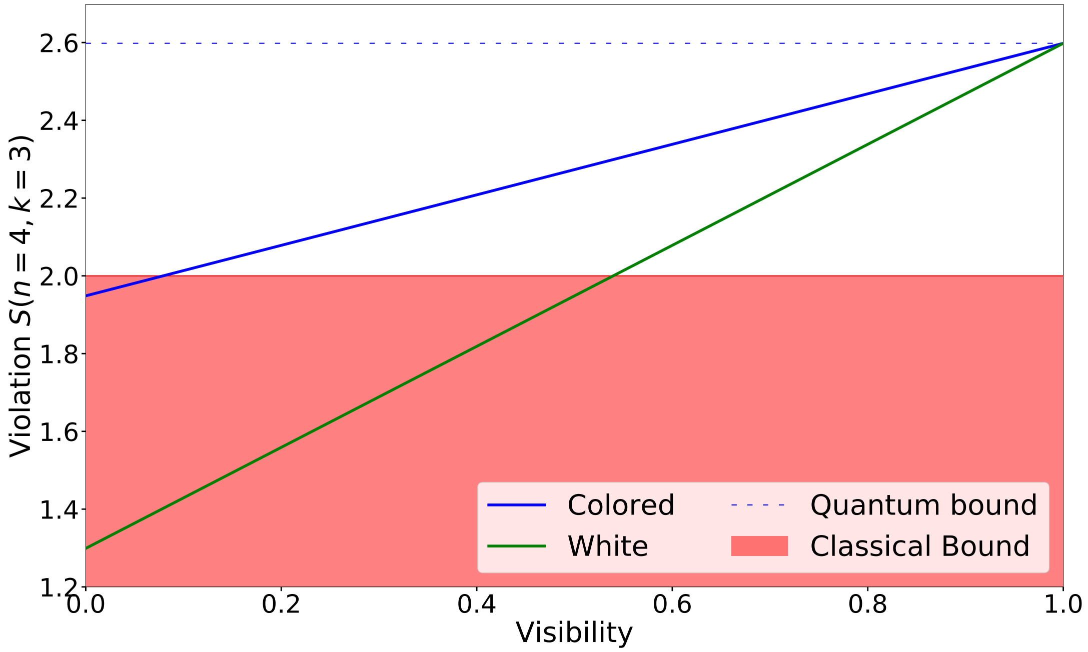

Supplementary Figure 6: **Comparison of different noise type affecting S:** Plot of the simulation results for the S value in a 4-points star-network, where each agent performs $k = 3$ measurements. As the visibility decreases, the violation S drops from the quantum to the classical bound. The blue curve refers to violation against coloured noise, while the green one to violation against white noise. We observe that the amount of violation is more sensitive to white noise.

B. Laboratory efficiency estimation

We tested the stability of each entangled source, as a mean to monitor the efficiency of the overall system. Through CHSH inequality violations, we certified the amount of entanglement for each pair of photons, together with the visibility of each source. In particular, the CHSH inequalities were tested by measuring one photon in the corresponding laboratory, while the other photon is sent and measured in the common station B. The extent of these CHSH violations are presented in Supplementary Table 1.

From these violations, we inferred the amount of noise affecting each source. For our generation method, it is known [4] that the white and colored noise affecting the generated pair are correlated. In particular, we assume the amount of white noise to double the colored one, i.e. $\lambda = \frac{1}{3}$ in Eq. (5).

Supplementary Note 3. DATA ANALISYS

A. Data acquisition

The data acquisition was performed by exploiting time-to-digital converters *id-Quantique ID800*, that allows to perform time tagging of all measured events, thus permitting to evaluate the number of coincidences. In detail, each event is represented by a pair of values: the label of the corresponding detector and its timestamp, i.e. the arrival time of the signal, with a resolution of ≈ 81 ps. In order to keep track of all the sources, five different coincidence counters were adopted, synchronizing them with a reference signal, shared by all of them. Such acquisition system allowed to test each laboratory individually and within the multipartite systems, i.e. bipartite, 3-partite and 4-partite.

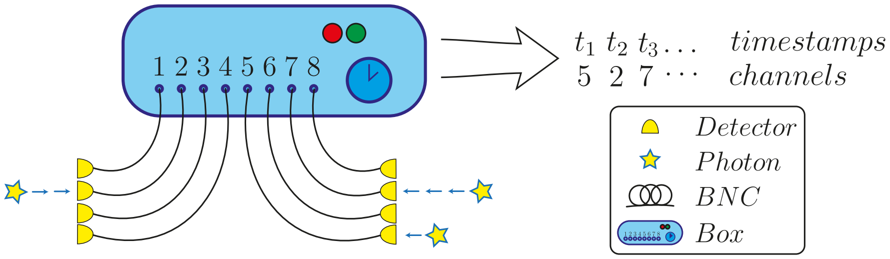

Supplementary Figure 7: **Coincidence counter scheme:** A scheme of the coincidence counter is depicted. It has two main functionalities: analog-to-digital conversion and a high precision chronometer. Thus, this device receives the analog signal of a photon detection (a star in the picture) from the single photon detectors via BNC cables and outputs digital data consisting of two arrays. One carries the events' timestamps and the other specifies from which input channel (detector) the signal came. The counter is an *id-Quantique ID800*.

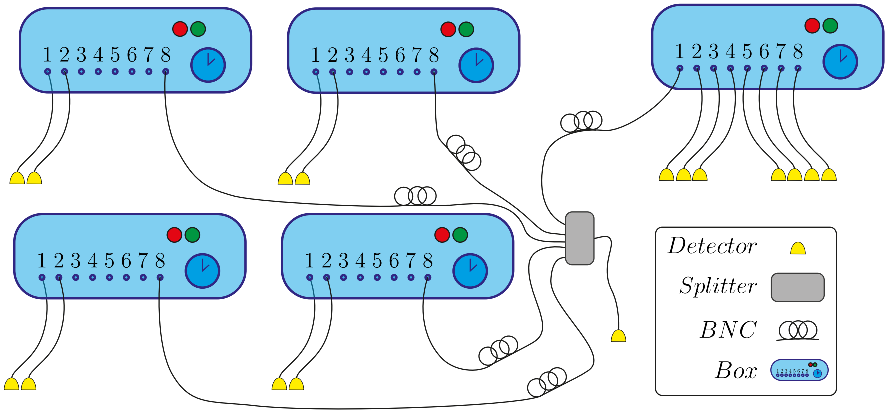

Supplementary Figure 8: **Configuration of the five coincidence apparatuses employed:** Five different coincidence apparatuses were employed to perform measurement of the 4-points star network. The apparatuses were located in different laboratories and were connected with different computers. A clock signal was sent to each system using a digital splitter. The output of one single photon detector of Bob's measurement station was used as clock signal shared by all the measurement systems.

B. Coincidences analysis

The software to coordinate the measurements among the laboratories was written in Python, and enabled communication between the laboratories using the local network. The data analysis was performed by a software written in C, for high performances and to deal with the large amount of data produced by the experiment. Indeed, due to the brightness of our sources, each second of acquisition produces about 7 MB of raw data. Hence, the overall amount of analysed data results in 400 GB.

1. *Two-fold coincidences*

Going into further details about the data recording and processing, each event detected by the *id-Quantique ID800*s counters was registered on a different file. Then, the files are analyzed in order to find all two-fold coincidence events. More specifically, a two-fold coincidence must satisfy two conditions: the events have to occur within a time interval of, at most, ≈ 3.24 ns and the pair must be compatible with the sources scheme. In other words, coincidence events corresponding to the source Λ_i must occur between the station of Laboratory i and the core's one, i.e. if we label with B_i the outcomes of the core station corresponding to the outlying agent A_i, only the pair $(B_i = \pm 1, A_i = \pm 1)$ are coherent with the sources' scheme (see Supplementary Figure 9). These two conditions are quite strict, indeed to have an idea of how tight is the first one, we observe that the detector dead-time is nominally 22 ns, that is, approximately 7 times the adopted time window. These requirements cut off most of the noise from the dark counts affecting any detector.

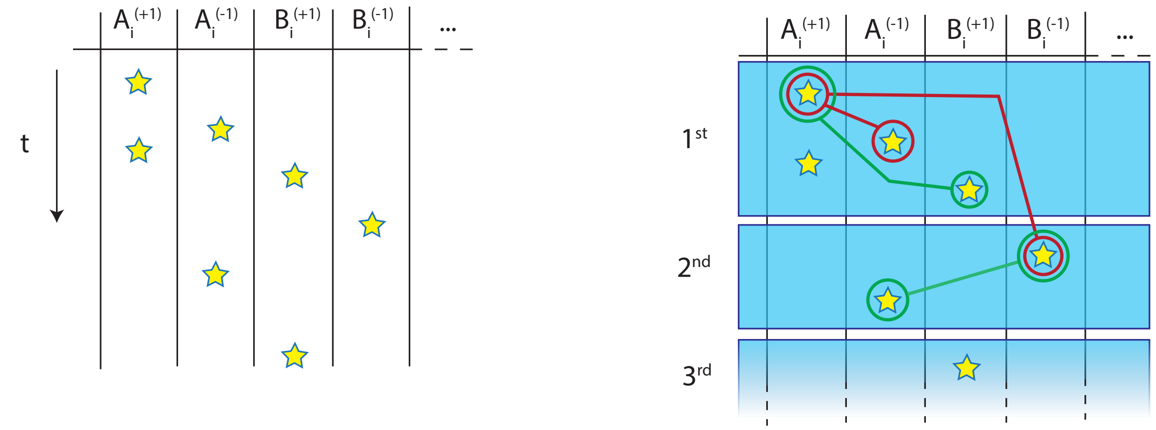

(a) Raw list of events.

(b) The way events are processed.

Supplementary Figure 9: **Structure of detection events provided by the coincidence apparatuses and their elaboration:** Each column represents a detector and thus corresponds to one of the two outcomes of the outlying agents A_i. A star in the column is a click of the corresponding detector. The vertical direction represents the time of each detections. **(a)** A pictorial scheme of how the events are recorded. **(b)** Scheme of the processing algorithm. The green-circled events are the pairs that respect the two-fold conditions. The red-circled ones are events that are discarded since either they do not satisfy the apparatus scheme or they occur at a distance larger than a window width ($w = 3.24$ ns). In this case, the resulting two-fold events are: $(A_i^{(+)}, B_i^{(+)})$ and $(A_i^{(-)}, B_i^{(-)})$. The cardinal numbers on the left identify different windows, whose width represents the time interval within which the corresponding coincidence event is recorded, which is upper-bounded by w.

2. *n-fold coincidences*

Moreover, the list of two-fold coincidences obtained as described above has to be further analysed to extract coincidence between different branches of the star-network, i.e. obtaining n-fold events. A n-fold event is a set of two-fold events that can be considered synchronous for another given window different from the one used to extract two-fold coincidences.

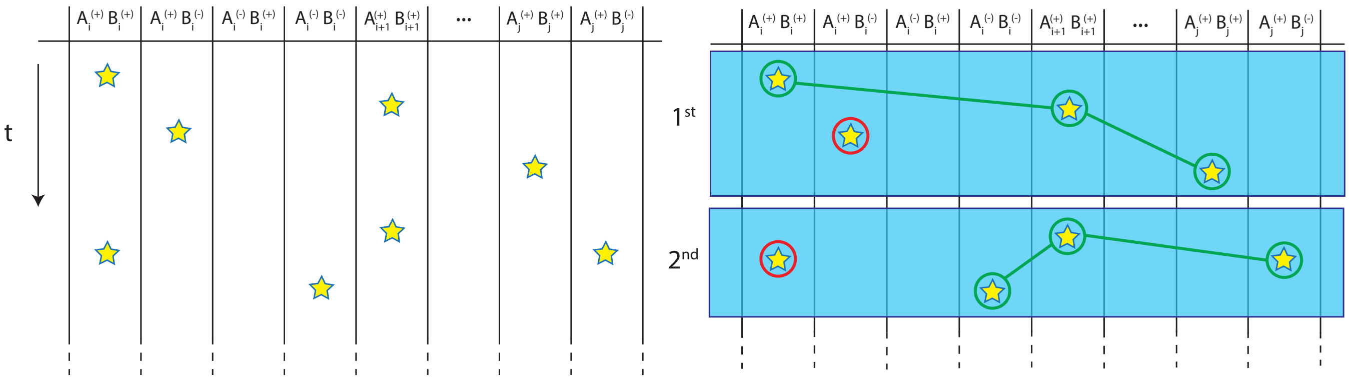

(a) List of two-fold events.

(b) The way two-fold events are processed.

Supplementary Figure 10: **Structure of two-fold events retrieved by the previous analysis and their elaboration:** Each column represents a pair of detectors. A star in the column is an occurrence of that pair. The vertical direction qualitatively represents the time of each detection. **(a)** A pictorial scheme of how the events result from the previous analysis. **(b)** Scheme of the processing algorithm. The cardinal numbers on left identify different windows. The green-circled events are the pairs that respect the two-fold conditions. The red-circled ones are events that are discarded, since either they do not satisfy the apparatus scheme or they occur at a distance larger than the maximal window width chosen for n-fold events. The width of the first window depicted is wider than the second since the chosen value is only an upper bound. The resulting six-fold events are: $(A_i^+ B_i^+\ , A_{i+1}^+ B_{i+1}^+\ , A_j^+ B_j^+)$ and $(A_i^- B_i^-\ , A_{i+1}^+ B_{i+1}^+\ , A_j^+ B_j^-)$.

C. Coincidences window

To better understand the role of the coincidence window, we will exploit the *triggered coincidences* method as a guiding idea. The first detection event of the list opens a *window* that defines the maximum time interval within which the measured events are considered simultaneous. The analysis software now only looks for events from the other detectors in order to find one of the possible n-fold configuration respecting the source configuration. Subsequent events registered by the same detector will not be counted. As soon as all the conditions defining a n-fold event are met, or the window expires, the software stops looking and a new window will be opened by the next occurring event. The aforementioned data processing does not bias the event statistics since it does not favor any measurement outcome amongst the others.

The adopted strategy for the definition of the n-fold window corresponds to choosing the time interval maximizing the violation, i.e. the number of standard deviations between the experimental value of S_n^k and its classical bound $B^{\mathrm{C}}(n,k) = k-1$. A wider window implies a less strict condition for considering events as simultaneous which increases the number of n-fold coincidences, and improves the statistical error estimated assuming a Poissonian distribution for the events. Hence, it is not surprising that the optimal window is the widest one considered ($\approx 81\mu$s) which, for example, gives a violation of $\approx 23\sigma$ for S_4^2. Nonetheless a significant violation for S_n^k, can be obtained for several values of the window, ranging from 0.49 μs to 81 μs. The plot in Supplementary Figure 12 shows the expected behaviour of a decreasing and stabilizing violation as the number of counts increase. In particular, for example, for the smallest window considered (0.49 μs) we still have $S_4^2 = 1.209 \pm 0.016$ which gives a violation of $\approx 12.5\sigma$.

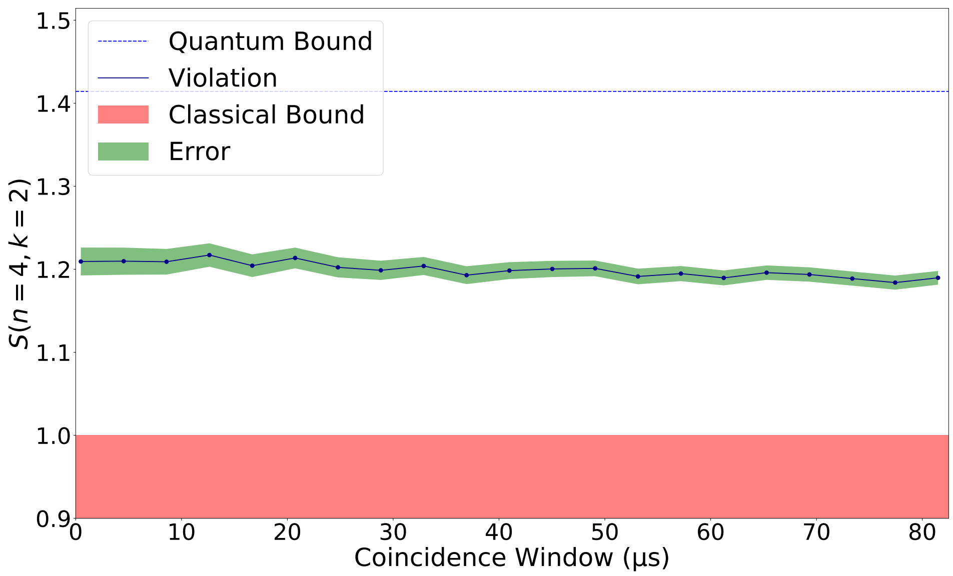

Supplementary Figure 11: **Signal for violation as a function of the n-fold coincidence window length:** The n-fold coincidences identified by our software depend on the choice of time window within which events are considered as simultaneous. In order to choose the best value for this time interval and to check how this value influences the signal for violation, we analyzed $S(n,k)$ as a function of the window. In the plot we are showing the violation in the case $n=4, k=2$, evaluated with the data from a multiple exposures for a total time of 34.2 s. The blue line is the experimental value of $S(4,2)$. The green region represents the Poissonian error of the expected violation computed from the number of two-fold coincidences observed. The blue dashed line is the quantum bound while the red region is the value of $S(4,2)$ achievable within a classical model.

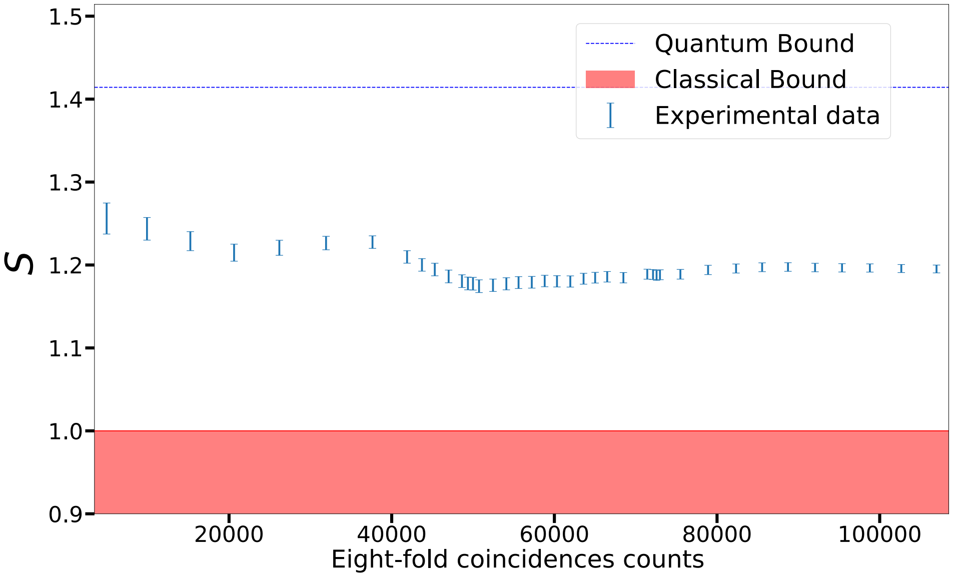

Supplementary Figure 12: **Graph of the signal for violation as a function of the number of tuple events** The blue scatter plot represents the value of S_4^2 computed upon an increasing statistical sample quantified by the number of measured n-fold events. The n-fold window width is fixed at 80 μs, the counts growth is due to an greater overall exposition time considered. The red region corresponds to the value of S achievable with classical correlations. Error bars represents the Poissonian error on S.

SUPPLEMENTARY REFERENCES

[1] Eisaman, M. D., Fan, J., Migdall, A., Polyakov, S. V. Invited review article: Single-photon sources and detectors. *Review of scientific instruments* **82**, 071101 (2011).

[2] Kim, T., Fiorentino, M., Wong, F. N. Phase-stable source of polarization-entangled photons using a polarization sagnac interferometer. *Physical Review A* **73**, 012316 (2006).

[3] Fedrizzi, A., Herbst, T., Poppe, A., Jennewein, T., Zeilinger, A. A wavelength-tunable fiber-coupled source of narrowband entangled photons. *Optics Express* **15**, 15377–153 (2007).

[4] Cabello, A., Feito, Á., Lamas-Linares, A. Bell's inequalities with realistic noise for polarization-entangled photons. *Phys. Rev. A* **72**, 052112 (2005).

[5] Carvacho, G., Andreoli, F., Santodonato, L., Bentivegna, M., Chaves, R., Sciarrino F. Experimental violation of local causality in a quantum network. *Nature communications* **8**, 14775 (2017).
